# The deletion of M_4_ muscarinic receptors increases motor activity in females in the dark phase

**DOI:** 10.1002/brb3.1057

**Published:** 2018-07-06

**Authors:** Paulina Valuskova, Sandor T. Forczek, Vladimir Farar, Jaromir Myslivecek

**Affiliations:** ^1^ 1st Faculty of Medicine Institute of Physiology Charles University Prague Czech Republic; ^2^ Isotope Laboratory Institute of Experimental Botany Academy of Sciences of the Czech Republic Prague Czech Republic

**Keywords:** biorhythm, intergeniculate leaflet, M_4_ muscarinic receptor, motor activity, motor cortex, sex differences, striatum, suprachiasmatic nuclei, temperature, thalamus

## Abstract

**Objectives:**

M_4_ muscarinic receptors (MR) presumably play a role in motor coordination. Previous studies have shown different results depending on genetic background and number of backcrosses. However, no attention has been given to biorhythms.

**Material and Methods:**

We therefore analyzed biorhythms under a light/dark cycle obtained telemetrically in intact animals (activity, body temperature) in M_4_ KO mice growth on the C57Bl6 background using ChronosFit software. Studying pure effects of gene knockout in daily rhythms is especially important knowledge for pharmacological/behavioral studies in which drugs are usually tested in the morning.

**Results:**

We show that M_4_ KO mice motor activity does not differ substantially from wild‐type mice during light period while in the dark phase (mice active part of the day), the M_4_ KO mice reveal biorhythm changes in many parameters. Moreover, these differences are sex‐dependent and are evident in females only. Mesor, night–day difference, and night value were doubled or tripled when comparing female KO versus male KO. Our in vitro autoradiography demonstrates that M_4_ MR proportion represents 24% in the motor cortex (MOCx), 30% in the somatosensory cortex, 50% in the striatum, 69% in the thalamus, and 48% in the intergeniculate leaflet (IGL). The M_4_ MR densities were negligible in the subparaventricular zone, the posterior hypothalamic area, and in the suprachiasmatic nuclei.

**Conclusions:**

We conclude that cholinergic signaling at M_4_ MR in brain structures such as striatum, MOCx, and probably with the important participation of IGL significantly control motor activity biorhythm. Animal activity differs in the light and dark phases, which should be taken into consideration when interpreting the results.

## 1 INTRODUCTION

Muscarinic receptors (MR) are typical members of G protein‐coupled receptors (Kruse et al., 2013) and can be divided into five subtypes (M_1_–M_5_), which activate different G proteins (G_q_, G_i_) (Eglen, 2012; Kow & Nathanson, 2012; Reiner & Nathanson, 2012). The often‐overlapping pattern of MR subtype expression and the lack of highly selective ligands toward a given MR subtype have precluded the precise delineation of MR subtype‐specific roles. To overcome this issue, gene targeting strategies have been employed and knockout mice for each MR subtype were generated and have been intensively studied (Wess et al., 2003). However, often contradictory results have been reported, particularly in terms of the role of M_4_ MR in the motor activity control. Moreover, the changes in motor activity have been usually demonstrated in a short stretch of time.

The initial knockout study (Gomeza et al., 1999) strongly indicated that M_4_ knockout significantly increases the overall animal motor activity. The increased locomotion of M_4_ KO mice has been attributed to the enhanced dopaminergic signaling at D_1_ dopamine receptors. Nevertheless, other M_4_ KO study in which backcrossing was carefully performed showed no M_4_ effects on motor activity (Woolley, Carter, Gartlon, Watson, & Dawson, 2009). A recent study in which a relatively long (30 min) evaluation of motor activity was performed showed an increase in motor activity (Koshimizu, Leiter, & Miyakawa, 2012). The initial studies were performed on mixed 129SvEv/CF‐1 background while Koshimizu et al. (2012) worked with animals made on a pure 129SvEv background.

Knockout studies were initially considered as an optimal method for detection of gene function (Bymaster, McKinzie, Felder, & Wess, 2003). However, the flanking allele effect was not sometimes considered as an important factor for behavior determination (Crusio, Goldowitz, Holmes, & Wolfer, 2009). It is also necessary to stress that mice are nocturnal animals (Roedel, Storch, Holsboer, & Ohl, 2006), and thus, experiments performed in their nonactive phase can be affected by this fact.

It is sometimes difficult to compare the types of motor activity that are followed in different studies (open‐field locomotor activity in boxes or on plus mazes, circadian activity on running wheels, or in cages). In general, all these motor activities are directed by similar mechanisms, and thus, it could give us the picture of differences in motor activity between different groups of mice. It has been shown previously that different types of locomotor activity are affected by sex steroid hormones. There were found differences in open field (Blizard, Lippman, & Chen, 1975), circadian genes expression (Kuljis et al., 2013), open field, light‐dark transition test, running wheel, and elevated plus maze (Morgan & Pfaff, 2001). Concerning the mechanisms, female sex steroid (estrogen) has been shown to increase locomotor activity (Ogawa, Chan, Gustafsson, Korach, & Pfaff, 2003) and in open field (Morgan & Pfaff, 2001). Thus, we expected differences between males and females.

It seems that multiple brain areas drive biorhythmic coordination in locomotor activity (Myslivecek, Farar, & Valuskova, 2017). The most prominent structure is, of course, the suprachiasmatic nucleus (SCN). Other structures have been also implicated in these effects. There are areas with near proximity to SCN, such as the subparaventricular zone (SPVZ), the dorsomedial nucleus, and the posterior hypothalamic area (PHA) and the tuberomammillary nucleus (Abrahamson & Moore, 2006; Kramer et al., 2001). The striatum, the thalamus, and the intergeniculate leaflet (IGL (Hughes & Piggins, 2012; Morin, 2013)) are also areas with locomotor biorhythmic effects. The SCN is innervated by cholinergic nerves (Hut & Van der Zee, 2011), but does not need to be necessarily intrinsically cholinergic (van den Pol & Tsujimoto, 1985). It receives cholinergic projections from basal forebrain and brain stem tegmentum (Bina, Rusak, & Semba, 1993). There are species differences in the presence of cholinergic neurons in the SCN in rat, hamster, and mouse (Hut & Van der Zee, 2011).

We, therefore, studied activity and body temperature biorhythm under a light/dark cycle in well‐defined C57BL/6 mice and in their counterparts lacking M_4_ MR using a telemetric system that allowed us to see the pure knockout effect without the influence of handling or other manipulation. In addition to that this model can also show the effect of knockout on clear genetic background (see flanking allele effect described above). Studying pure effects in biorhythms is especially important knowledge for pharmacological and/or behavioral studies in which drugs/treatment or tests are usually performed in the morning (i.e., in the nonactive phase in mice).

We tested the hypothesis that M_4_ MR affect the animal activity without an effect on body temperature. The basis for this comes from previously published data about M_4_ KO mice that elicit similar hypothermic response as wild types (Bymaster et al., 2001). Moreover, we hypothesized that this effect can be seen in the dark period only (active part of the day), and, thus, the biorhythm characteristics would be changed accordingly. This hypothesis is based on the fact that M_4_ MR are considered as receptors able to inhibit acetylcholine release (Bymaster et al., 2003). Acetylcholine levels are higher in the active period (Hut & Van der Zee, 2011). Thus, the lack of inhibitory M_4_ MR would increase acetylcholine levels and, thus, increase locomotion in dark period. Last, we hypothesized that this difference is sexually dependent, because it has been previously shown that locomotor activity is affected by sex steroid hormones (see above).

One of the important questions in motor coordination regulation is the role of brain areas previously identified as connected with biorhythm regulation. Thus, we have performed autoradiography experiments and we compared MR density in several brain areas (motor cortex [MOCx], somatosensory cortex [SSCx], striatum, thalamus, IGL, SCN, SPVZ, and PHA) in WT and M_4_ KO mice. Binding in KO mice can supply us with data on the proportion of M_4_ MR. If the M_4_ MR gene is deleted, then the decrease in nonspecific MR ligand binding is equal to the proportion of M_4_ MR. If there is no decrease in binding, then no M_4_ MR are present, and it is unlikely that this brain area can be involved in events caused by M_4_ MR.

## 2 METHODS

### 2.1 Animals

The mice lacking the M_4_ muscarinic receptor were generated in Wess’ laboratory (Gomeza et al., 1999) and then bred in our animal facility (Prague, Czech Republic). Their genetic background was C57Bl6/6NTac. Animals were treated in accordance with the legislature of the Czech Republic and the EU legislature (European Convention for the Protection of Vertebrate Animals used for Experimental and other Scientific Purposes [Council of Europe No. 123, Strasbourg 1985]), and the experimental protocol was approved by the Committee for the Protection of Experimental Animals of the 1st Medical Faculty, Charles University, Prague, and by the Ministry of Education of the Czech Republic under No. MSMT‐2409/2017‐3. The wild‐type line was C57BL/6NTac line. We studied fully backcrossed (12 generations) muscarinic M_4_^−/−^ and M_4_^+/+^ littermates. The animals were maintained under controlled environmental conditions (12/12 light/dark cycle, 22 ± 1°C, light on at 6:00 am). Food and water were available ad libitum. A total of 60 animals were used in the study: males (weighing 25–33 g, age 3–6 months) and females (weighing 20–26 g, age 3–6 months), of which there were 28 M_4_ KO animals (15 males, 13 females) and 32 WT (15 males, 17 females). Prior to the experiments, the mice were genotyped and only homozygous mice were used in the study. The females were housed separately from males and, thus, revealing Lee–Boot effect (i.e., suppressed estrus cycle—anoestrous) (Ma, Miao, & Novotny, 1998) which made the female group homogenous in hormone levels. Moreover, no differences were seen in actograms in females during 15 consecutive days.

### 2.2 Telemetry

In order to judge the biorhythm changes in intact animals, we employed a telemetric apparatus to measure body temperature and overall motor activity. The telemetry system used was commercially available from Mini Mitter (Starr Life Sciences Corp., Oakmont, PA, USA, originally from Respironics, Andover, MA, USA). The transponders (E‐Mitter, G2, length 15.5 mm, 1.1 g) were implanted in the peritoneal cavity under the anesthesia (Zoletil^®^ 100, Rometar^®^ 2% 5:1, diluted 10 times, 3.2 ml/kg). During the implantation, the mice were kept on the thermostable pad. Mice were left 1 week for recovery from the surgery and then used in the experiment. The temperature and activity were acquired directly from the transponders in the sample period for three consecutive days during which the animals were not disturbed. Similar rhythms were recorded before and after this sample period. The temperature and activity were recorded in home cages of typical size (38 × 22 × 15 cm). Receivers were connected in series and connected directly to the PC into a single computer port, allowing for the determination of all parameters. The data were collected every 60 s. VitalView software was used for the acquisition and first processing of data.

### 2.3 Biorhythm analysis

The data collected by telemetry were grouped into 10‐min sequences, and the calculated means were used for further analysis. The analysis was performed using the ChronosFit program (Arraj & Lemmer, 2006) employing Fourier analysis and the stepwise regression technique. Then, the data were transferred into the GraphPad Prism 5.04 program (San Diego, USA) for further statistical analysis.

### 2.4 Receptor autoradiography

For receptor determination, autoradiography was performed in several brain areas (MOCx, SSCx, striatum [CPu], thalamus [TH], SCN, SPVZ, PHA, and IGL). Brains were rapidly removed (four to six brains per group), frozen in dry ice, and then stored at −80°C until cryostat sectioning. Sixteen‐micrometer‐thick sagittal or frontal sections were cut on a cryostat at −20°C and thaw‐mounted on Superfrost^®^ Plus glass slides (Carl Roth GmbH & Co. KG, Karlsruhe, Germany) and stored in storage boxes at −80°C until use. For binding to MR, the sections were allowed to thaw and dry for 30 min at 22°C and the density of receptors was determined as previously described (Farar & Myslivecek, 2016; Farar et al., 2012; Valuskova, Farar, Forczek, Krizova, & Myslivecek, 2018). In brief, sections were incubated for 2 h with 2 nM [^3^H]‐QNB at room temperature. Nonspecific binding was assessed on adjacent sections in the presence of 10 μM atropine sulfate. After incubation, the sections were washed two times for 5 min and gently dried. Dry sections were apposed to the tritium‐sensitive Fuji BAS imaging plates (GE Healthcare Europe GmbH, Freiburg, Germany) in Kodak BioMax autoradiographic cassettes (Carestream Health, Inc., Rochester, NY) for 5 days. The linearity of the signal and conversion of photostimulated luminescence to radioactivity was assessed using tritium autoradiographic standards (American Radiolabeled Chemicals, Inc., St. Louis, MO) The film autoradiograms were scanned, and the densitometry was performed with PC‐based analytical software, MCID analysis software. Measurements were taken and averaged from at least three sections for each animal and brain region.

### 2.5 Histology

Nissl staining was used for SCN, SPVZ, IGL, and PHA identification in MR autoradiography determination. In brief, the parallel sections were obtained using cryostat (the appropriateness of section was controlled using mice atlas (Paxinos & Franklin, 2008)), the sections were collected and divided into four sets. The first section from the set was placed on the first glass slide and used for Nissl staining, while the remaining four sections from the set were placed on other glass slides (three sections from different sets on one glass slide) and used for autoradiography. The sections used for Nissl staining were immersed in a solution of alcohol (70%, 80%, 96%) for 2 min each, stained with Nissl solution (1% cresyl violet and 0.2 mol/L acetic acid+ 0.2 mol/L sodium acetate, 4:1, pH = 3) for 20 min, then twice washed in distilled water and immersed in a solution of alcohol (96%, 80%, 70%) for 2 min each. Then, the samples were immersed into xylene (xylene, mixture of isomers, p.a., Penta, Czech Republic) for 5 min. Then, the sections were incubated for another 45 min in xylene (p.a., Penta, Czech Republic) and mounted using DPX (Sigma‐Aldrich, Czech Republic) with a coverslip.

The area, clearly visible as in Nissl staining, was then marked (using border transposition) on a scanned autoradiogram and used for densitometry with PC‐based analytical software (MCID software).

### 2.6 Statistical analysis

As some variables from biorhythm analysis revealed dependency as also verified by Pearson's *r* (close to unity), we have used one‐way ANOVA for analysis with post hoc Sidak's corrections. Values of *p* < 0.05 were considered significant. If the variables were independent, then there was statistical significance between the groups determined using the Student *t* test (WT vs. KO animals). In the same way, *p* < 0.05 was considered significant.

## 3 RESULTS

### 3.1 Activity

#### 3.1.1 Males

**Figure 1:**
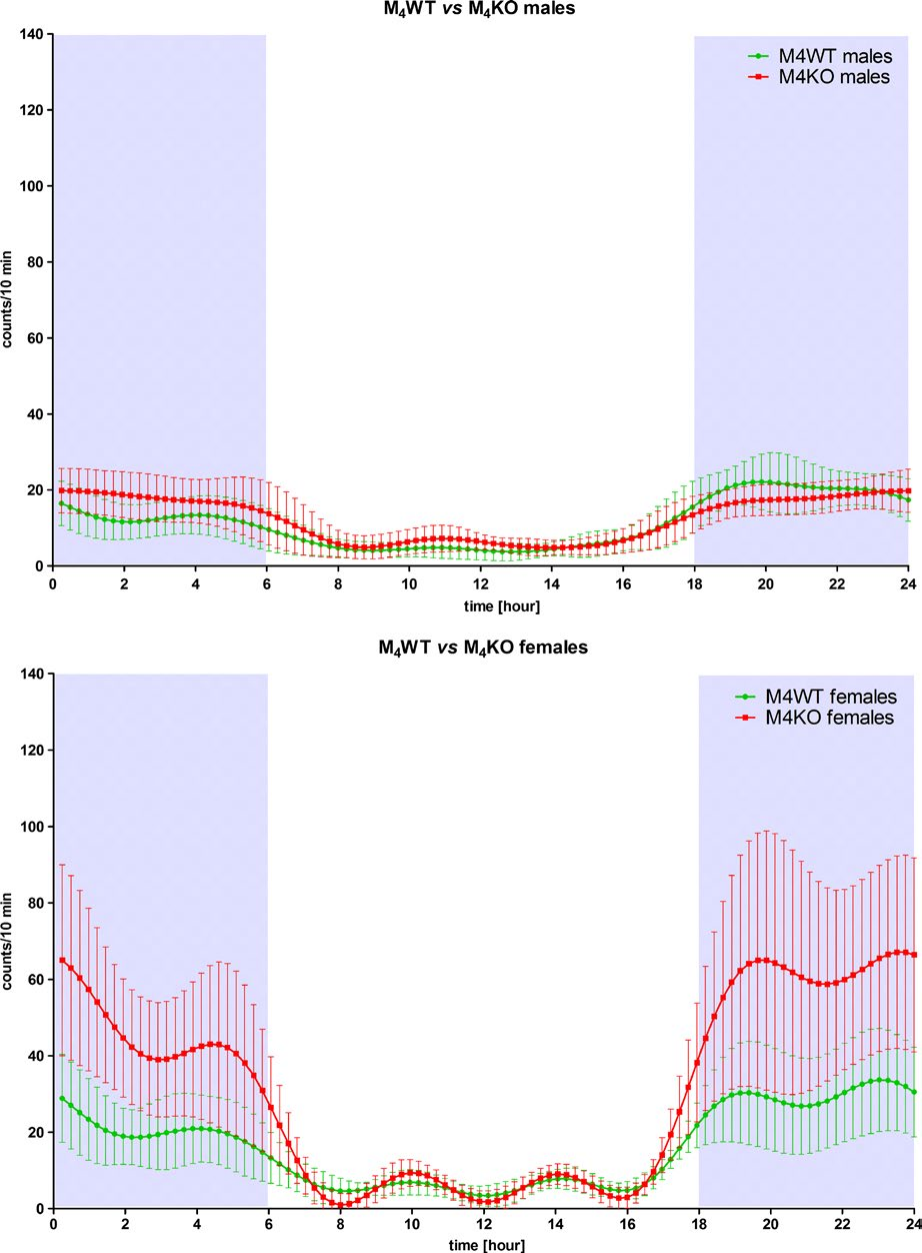
Biorhythm of locomotor activity in males (above) and in females, showing various curves in WT and KO animals. Activity was measured using telemetry, and mean and *SD* are shown. See legend for symbol explanation

The biorhythm in M_4_ KO was changed only in a minor manner (as can be seen from Figure 1) in comparison with control animals (WT mice, see Table 1, ANOVA: *F*_7,112_ = 25.39, *p* < 0.0004). Dominating period length was 24 hr in both groups. Both strains, WT and KO, reveal typical pattern for nocturnal animals in activity (ACT) and temperature (TEMP) with peak values in the dark period.

**Table 1:** Rhythm analyses of ACT (motor activity), and TEMP (temperature) in WT and M_4_ KO mice males and females

	ACT				TEMP			
	Males		Females		Males		Females	
	Mean	*SEM*	Mean	*SEM*	Mean	*SEM*	Mean	*SEM*
WT								
Mesor	11.25	0.49	16.06#	1.02	36.17	0.07	36.86###	0.04
Day	6.10	0.26	6.94	0.47	35.69	0.07	36.26###	0.04
Night	16.40	0.98	25.58###	1.95	36.64	0.12	37.45###	0.05
N‐D	19.30	1.04	18.64###	1.85	0.95	0.11	1.19#	0.05
M_4_ KO								
Mesor	12.4	0.57	30.70**###	2.85	36.31	0.03	36.95###	0.05
Day	7.26	0.38	7.13	0.37	35.78	0.03	36.24###	0.06
Night	17.81	1.07	54.26***###	5.49	36.83	0.05	37.66###	0.08
N‐D	10.55	1.08	47.14***###	5.30	1.05	0.06	1.42*###	0.08

*Notes*

The mesor, nighttime (night), and daytime (day) mean values, including the night–day difference (N‐D), are shown. Data were analyzed using one‐way ANOVA with Sidak's test used for post hoc analysis. Data are expressed as mean±SEM.

*p < 0.05, ***p < 0.001: different from WT animals. #*p* < 0.05, *##*p* < 0.01, ###*p* < 0.001: different from males.

Also, these biorhythms had a similar pattern (See Supporting Information Table S1) obtained from ChronosFit analysis. The curves differed mainly in maximal and minimal slope which was much lower in KO (KO vs WT: *t*(28) = 4.021, *p* = 0.0004, 1.53% both in maximal and minimal slope). Also, there was slight increase in the highest day/night value in KO, that is, in D highest (*t*(28)  = 2.337, *p* = 0.027, 114%), N highest (*t*(28) = 2.657, *p* = 0.013, 110%), and in area under the curve (T AUC, ANOVA: *F*_25,364_ = 397.1, *p* < 0.0001, 110%). The period lengths (number of periods, 3.07 vs. 4.13 in KO and WT, respectively, *t*(28) = 2.486, *p* = 0.013) were slightly decreased in KO animals (to 74%), similarly, the percentage of 24‐hr rhythm (that was decreased to 71%, *t*(28) = 2.674, *p* = 0.0124).

#### 3.1.2 Females

In deep contrast to males (please compare curves shown in Figure 1 and data in Tables 1 and 2), M_4_ KO females revealed huge biorhythm changes that were mainly caused by increased nocturnal activity. Dominating period length was 24 hr in both groups. The mesor (a midline based on the distribution of values across the cycles of the circadian rhythm, computed using a cosine function, see Table 1) in KO animals was increased (ANOVA: *F*_7,112_ = 37.44, *p* < 0.0001) to 191% when compared to the values in WT animals. There was also an increase in KO in the night values (212% of WT value) and in the night–day difference (253% of WT value). Further parameters (ANOVA: *F*_25,364_ = 131.3, *p* < 0.0001), that is, T AUC [area under curve], AUC24 [AUC over exactly 24 hr], T highest [the highest value], N AUC [night area under curve], and N Highest [highest value measured in night period]) that have been changed in KO females are summarized in Table 2. Moreover, the amplitudes of 24‐hr 12‐hr 6‐hr, and 4.8‐hr rhythm were also doubled (or more than doubled, ANOVA: *F*_13,127_ = 6.573, *p* < 0.0001) in KO animals. Thus, we compared power spectrums in WT and KO animals (Supporting Information Figure S1) and find differences between these animals and in the power of the 24‐hr period (ANOVA: *F*_3,52_ = 23.88, *p* < 0.0001).

**Table 2:** Differences in activity biorhythm parameters between WT and M_4_KO females

Parameter	T AUC	AUC24	T Highest	D Highest
Significance	***	***	***	**
% KO versus WT	191.08	197.85	175.90	172.46
Parameter	N AUC	N Highest	Peak	Trough
Significance	***	***	***	*
% KO versus WT	217.98	175.90	205.90	−43.36
Parameter	Amp 24	Amp 12	Amp 6	Amp 4.8
Significance	***	*	**	***
% KO versus WT	238.61	204.90	223.97	196.44
Parameter	T AUC	AUC24	T Highest	D Highest
Significance	***	***	***	**
% KO versus WT	191.08	197.85	175.90	172.46
Parameter	N AUC	N Highest	Peak	Trough
Significance	***	***	***	*
% KO versus WT	217.98	175.90	205.90	−43.36
Parameter	Amp 24	Amp 12	Amp 6	Amp 4.8
Significance	***	*	**	***
% KO versus WT	238.61	204.90	223.97	196.44

*Notes*

One‐way ANOVA with post hoc Sidak's corrections or using Student *t* test in parameters that do not reveal correlations (WT vs. KO animals). T AUC, total area under curve; AUC24, area under curve in 24‐hr period; T Highest, the highest value; D Highest, highest value measured in day period; N AUC, area under curve in night period; N Highest, highest value measured in night period; Peak, the peak value calculated from the fitted curve; Trough, trough value calculated from the fitted curve; Amp 24, Amp 12, Amp 6, Amp 4.8, amplitudes of specific (24‐, 12‐, 6‐, 4.8‐hr) rhythms.

*p < 0.05, **p < 0.01, ***p < 0.001.

#### 3.1.3 Females versus males

**Figure 2:**
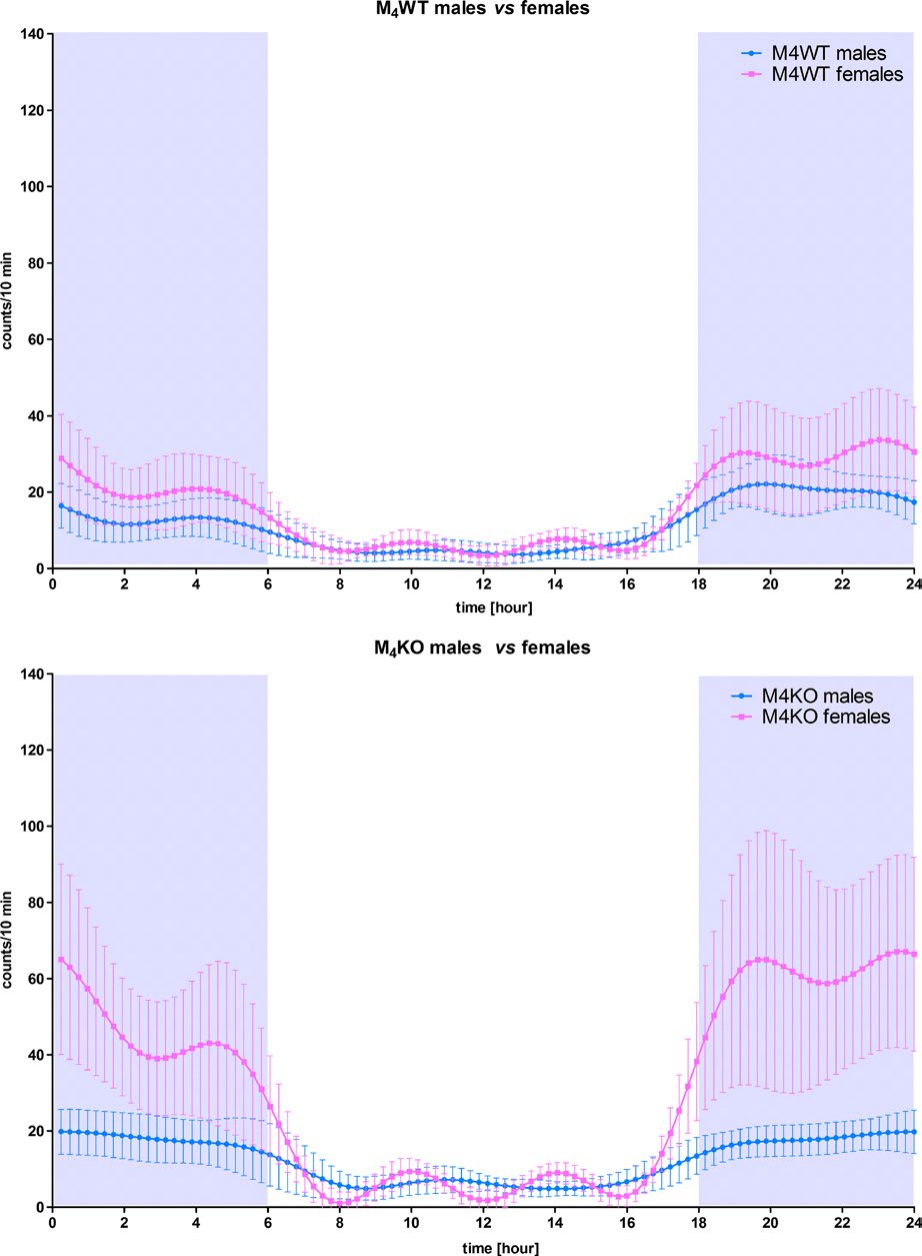
Comparison of different pattern of biorhythms in males and females. Top: males, bottom: females. Activity was measured using telemetry, and mean and *SD* are shown. See legend for symbol explanation

It can be seen from Figure 2 that there was a difference between female and male overall activity. This can be seen in WT animals (ANOVA: *F*_7,120_ = 29.98, *p* < 0.0001, see Tables 1 and 3), but to a much higher extent in KO animals (ANOVA: *F*_7,104_ = 44.02, *p* < 0.0001, see Tables 1 and 4). There were common differences: in mesor (which was 1.42 times higher in WT females and 2.47 in KO females, respectively), nighttime mean (1.56 increase in WT females, 3.05 in KO females, respectively), and difference between night mean and day mean (N‐D, 1.81 increase in WT females, 4.47 in KO females, respectively). In WT females (see Table 3), there were also increases (ANOVA: *F*_25,390_ = 226, *p* < 0.0001) in T AUC (1.42), AUC24 (1.41), N AUC (1.56), N Highest (1.34), and amplitudes (ANOVA: *F*_13,111_ = 2.974, *p* < 0.0009) of 24‐hr (1.61), 12‐hr (1.12), and 4‐hr rhythm (2.07).

**Table 3:** Differences in activity biorhythm parameters between WT males (M) and females (F)

Parameter	T AUC	AUC24	N AUC	N Highest
Significance	***	***	***	***
% F versus M	142.84	141.29	156.42	134.13
Parameter	Amp 24	Amp 12	Amp 4	
Significance	**	**	*	
% F versus M	161.40	111.83	207.99	

*Notes*

For explanation of codes, see legend to Table 2.

*p < 0.05, **p < 0.01, ***p < 0.001.

**Table 4:** Differences in activity biorhythm parameters between M_4_KO males and females

Parameter	T AUC	AUC24	T Highest	N AUC
Significance	***	***	***	***
% F versus M	247.46	253.69	209.04	304.71
Parameter	N Highest	p. lengths	Amp 24	Amp 6
Significance	***	**	***	**
% F versus M	311.26	140.47	388.58	279.28
Parameter	Amp 4.8	Amp 4		
Significance	**	**		
% F versus M	393.20	290.31		

*p < 0.05, **p < 0.01, ***p < 0.001.

As stated before, the changes in KO animals were found to a greater extent. In addition to higher increases (twofold or threefold, ANOVA: *F*_25,338_ = 127.3, *p* < 0.0001) in T AUC (2.47), AUC24 (2.54), N AUC (3.04), and N Highest (3.11), there was also an increase (ANOVA: *F*_13,94_ = 6.951, *p* < 0.0001) in the number of periods (period length; 1.4 times) and amplitudes in 24‐hr, 6‐hr, 4.8‐hr, and 4‐hr rhythm.

**Figure 3:**
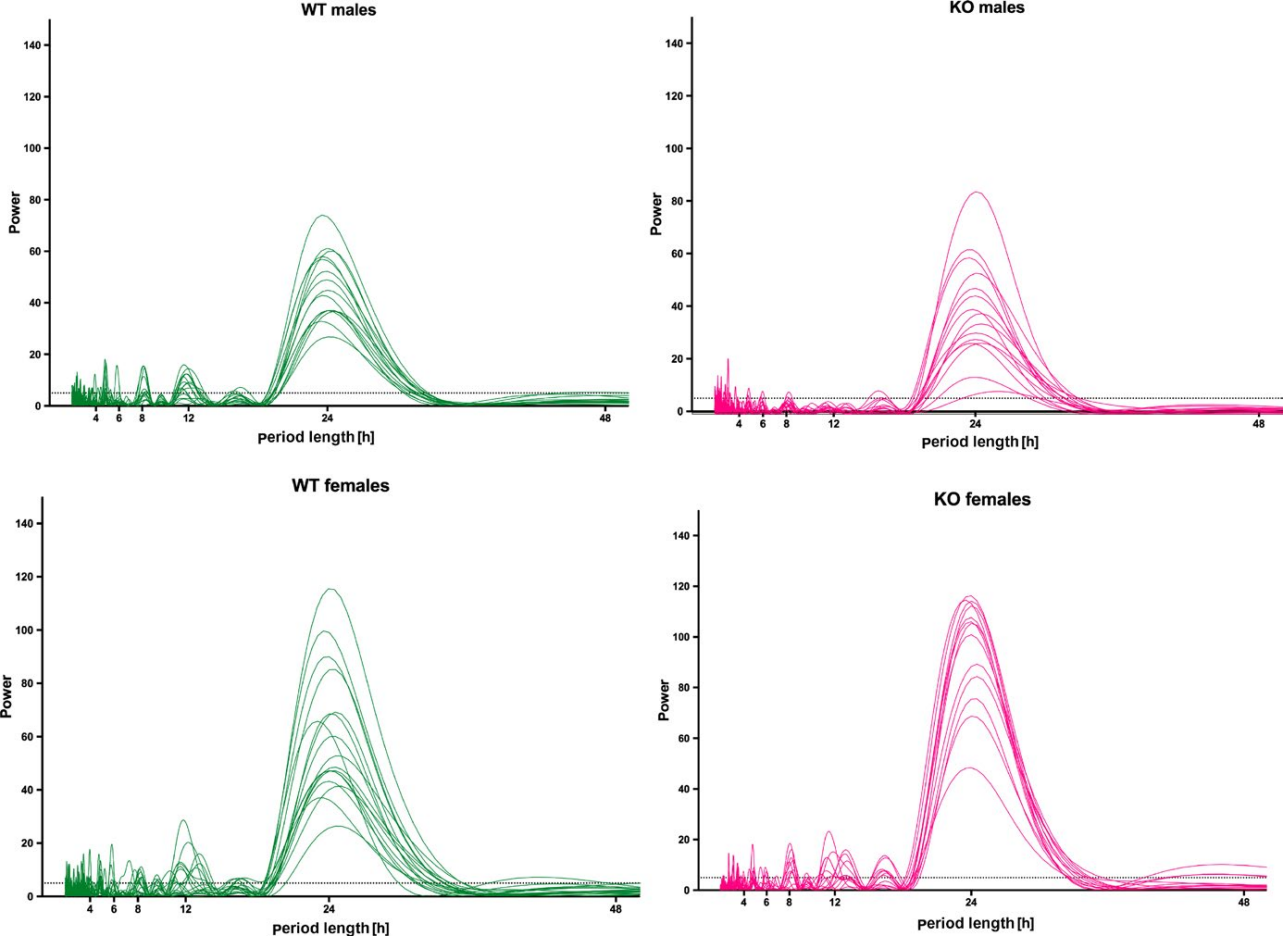
Comparison of activity periodograms in males and females. Left: periodograms in WT animals, males above, females bottom. Right: periodograms in KO animals, males above, females bottom. The spectrum was calculated by ChronosFit using Lomb–Scargle algorithm

Females also revealed higher power (ANOVA: *F*_3,52_ = 23.88, *p* < 0.0001) of the 24‐hr period when compared to males, as can be seen from periodograms shown in Figure 3.

### 3.2 Temperature

#### 3.2.1 Males

**Figure 4:**
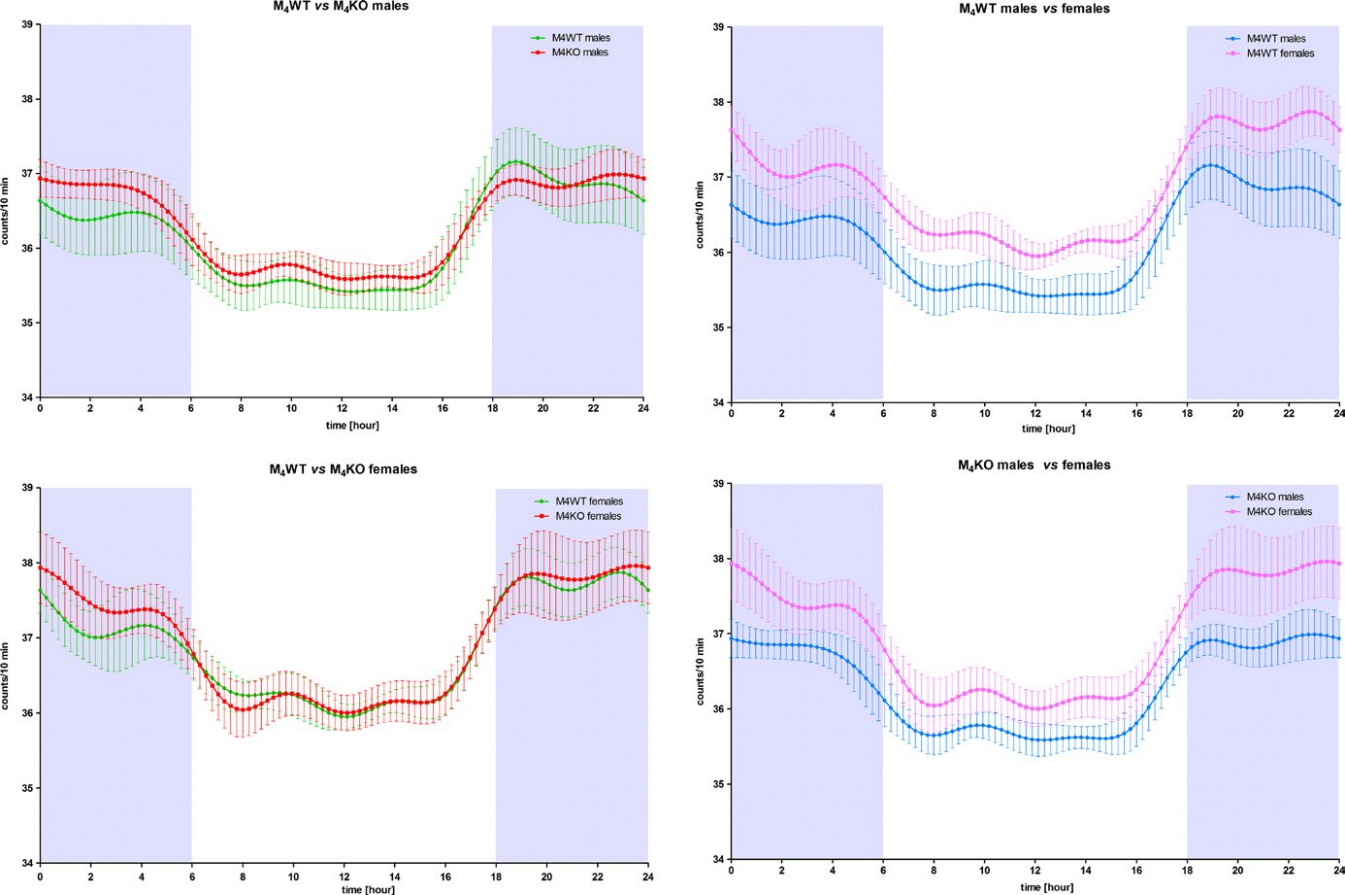
Left: Temperature biorhythm in males (above) and in females (bottom), showing various curves in WT and KO animals. Right: Comparison of biorhythms in WT (above) and KO (bottom) males and females. Temperature was measured using telemetry, and mean and *SD* are shown. See legend for symbol explanation

With an aim to determine whether M_4_ knockout specifically affects activity, we also followed the influence on body temperature. Figure 4 (left, above) shows that the temperature biorhythms were similar in WT and KO males. Only a few parameters (the lowest values in biorhythm curve [*t*(28) = 2.627, *p* < 0.0138] and amplitude in 12‐hr biorhythm [*t*(26) = 5.005, *p* < 0.0001]) were changed (see Supporting Information Table S2) and with only minimal difference.

#### 3.2.2 Females

Similar to males, only a few parameters differed (see Supporting Information Table S3) between WT and KO females (Figure 4 left, bottom) although the extent of changes was higher than in males: maximal slope (KO had this value increased to 340% of control, *t*(37) = 3.182, *p* = 0.003) and 12‐hr amplitude (KO had this value increased to 150% of control, *t*(30) = 2.274, *p* = 0.0303).

#### 3.2.3 Females versus males

It can be seen from Figure 4 (right above and bottom) that there was a slight increase in female compared to male temperature biorhythms. Although these increases were highly significant (ANOVA: *F*_25,429_ = 1434, *p* < 0.0001; *F*_13,95_ = 13.64, *p* < 0.0001), they were really small (see Supporting Information Tables S4 and S5) in units of percent (females vs. males) both in WT and KO animals.

### 3.3 **MR** density

**Figure 5:**
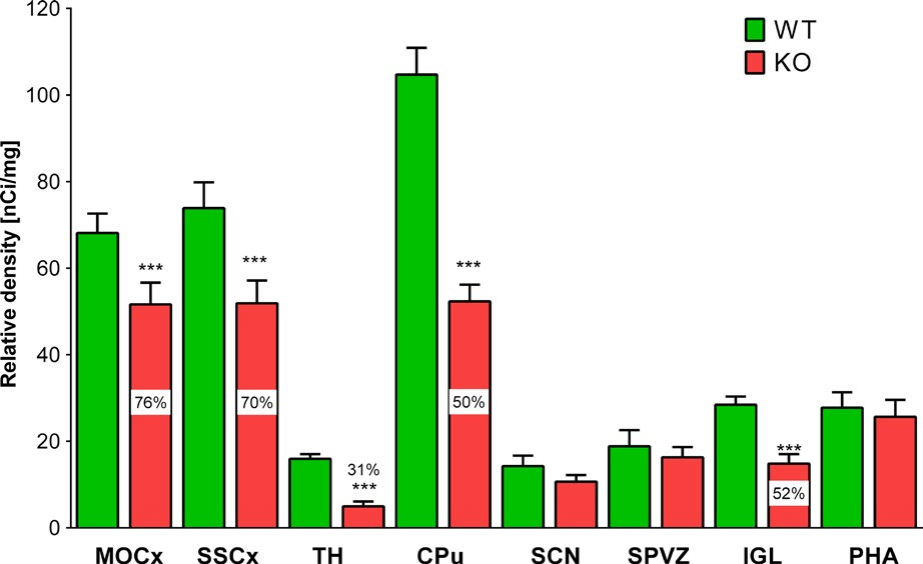
Relative density of muscarinic receptors (MR) in WT (WT) M_4_ KO (KO) mice in specific brain areas: motor cortex (MOCx), somatosensory cortex (SSCx), thalamus (TH) and caudatum‐putamen (CPu), suprachiasmatic nucleus (SCN), subparaventricular zone (SPVZ), intergeniculate leaflet (IGL), and posterior hypothalamic area (PHA). ***p < 0.001 difference from WT. Numbers in KO columns represent % of density in M_4_ KO mice

KO females showed decrease (Figure 5, ANOVA: *F*_15,72_ = 304.0, *p* = 0.0062) in MR density in the MOCx (to 76%, which means M_4_ MR represent 24%), SSCx (to 70%, i.e., 30% of M_4_ MR), striatum (to 50%, i.e., 50% of M_4_ MR), thalamus (to 31%, i.e., 69% of M_4_ MR), and in IGL (to 52%, i.e., 48% of M_4_ MR). No differences were seen in SCN, SPVZ, and PHA suggesting no M_4_ MR were present.

### 3.4 Histology

Representative sections comparing the histological and autoradiography picture are shown in Supporting Information Figure S2.

## 4 DISCUSSION

We demonstrate here that a lack of M_4_ MR increases motor activity in the dark period and this effect is much more pronounced in females than in males. These biorhythm changes were specific as another biorhythm—temperature—did not differ between animals with deleted M_4_ MR and control, that is, WT animals. Thus, there are no doubts about changed cholinergic signaling when M_4_ MR are deleted as reported multiple times (Bymaster et al., 2001, 2003; Wess et al., 2003).

These findings are, to our knowledge, new.

We used entrained rhythms under a light/dark cycle, and, as we have noticed above, some biorhythm parameters reveal dependency, which was taken into account in our analysis. However, there are also some mutually interconnected parameters, which should be mentioned, like higher activity during the dark period that will result in higher power in the 24‐hr period, which was found when comparing females to males.

Compelling evidence suggests an important role of the cholinergic system in the control of locomotor activity (Beeri et al., 1995; Martins‐Silva et al., 2011; Miyakawa, Yamada, Duttaroy, & Wess, 2001; Shapovalova, Kamkina, & Mysovskii, 2005). However, the role of M_4_ receptors in the control of locomotor activity remains elusive.

The initial generation of M_4_ KO mice linked M_4_ MR to the motor control. However, subsequent studies brought inconsistent and often contradictory results in terms of the involvement of M_4_ MR in motor control. This fact can be ascribed to the differences in several factors, including the genetic background of M_4_ KO mice, the method of locomotor activity assessment, the timing of experiments, and the gender used in the studies. It is important to note that the genetic background of animals in another cholinergic molecule (acetylcholinesterase knockout) causes different phenotypes in these mice (Duysen & Lockridge, 2006).

M_4_ KO mice (both sexes, maintained on mixed 129SvEv/CF1 genetic background) reported by Gomeza et al. (1999) were hyperactive in an open field during the first 40 min. By contrast, M_4_ KO male mice on pure 129SvEv background present increased locomotor activity only in the first 10 min (Koshimizu et al., 2012), and M_4_ KO males fully backcrossed to the C57BL/6NTac genetic background do not differ in open‐field locomotor activity from wild‐type controls, in spite of altered dopaminergic responses (Fink‐Jensen et al., 2011). Moreover, M_4_ KO males backcrossed to C57BL/6NTac genetic background do not differ with controls in the amount and diurnal pattern of sleep, locomotor activity, and temperature (Turner, Hughes, & Toth, 2010). To assess locomotor activity in undisturbed mice over a prolonged period (Turner et al., 2010), as we did, we employed a telemetric system. Consistent with this report, we did not find marked differences in M_4_ KO males. However, females, while not tested by Turner et al. (2010), in our study demonstrated increase in locomotor activity in their active phase only. Except for the work of Turner et al. (2010), all other reports tested locomotor activity only during the light period, which is the inactive phase of mice. It can be speculated about whether differences might be seen under the same experimental conditions (locomotor activity testing apparatus, gender, genetic background), but in the active phase of mice. The importance of experimental conditions, such as timing, in addition to genetic background, can be illustrated also by a different response of M_4_ KO to drug‐induced catalepsy. The work of Karasawa, Taketo, & Matsui (2003) indicated that M_4_ KO mice do not differ in haloperidol‐induced cataleptic responses, measured 30 min postinjection. In agreement with Karasawa et al. (2003) and Fink‐Jensen et al. (2011) reported the same cataleptic responses in M_4_ KO mice after 30 min, but marked attenuation of haloperidol‐induced catalepsy in M_4_ KO mice after 60 and 90 min, while administering the same dose of haloperidol as Karasawa et al. (2003). Moreover, Fink‐Jensen et al. (2011) used fully backcrossed mice. Consistent with Woolley et al. (2009), who used mice of unspecified sex (backcrossed for 10 +  generations), M_4_ KO males extensively backcrossed (for 11 generations, founders were mixed 129SvEv/CF‐1 background) to the C57BL/6NTac strain showed similar basal locomotor activity as their wild‐type counterparts (Schmidt et al., 2011).

In general, locomotor activity affects body temperature to some degree. However, while the activity was changed at least by one half, the temperature was not changed or changed to a minor extent only (compare data in Tables 1, 3, 4, and Supporting Information Tables S4 and S5). It is therefore probable that the M_4_ MR effect is specific to activity but not to temperature that is directed by other MR (at least partly, i.e., in hypothermic response), by M_2_ muscarinic subtype (Wess et al., 2003).

We have found a brain area‐specific decrease in MR using nonspecific radioligand ^3^H‐QNB, which depicts the M_4_ MR proportion in such a specific area. In other words, if there is 25% decrease in ^3^H‐QNB binding, then M_4_ MR represent 25% of total MR population. In SCN, the key structure governing circadian rhythms, we have found no changes in MR density in SCN in M_4_ KO animals, indicating only an inappreciable number of M_4_ MR. Thus, the density of M_4_ MR here is very low. In other brain regions was the proportion of M_4_ MR higher: Here, we describe about a one‐fourth decrease in the motor and somatosensory cortices (i.e., there is about 25% of M_4_ MR), about a one‐half decrease in the striatum (i.e., there is about 50% of M_4_ MR), two‐third decrease in the thalamus (about 30% of M_4_ MR), and about one‐half decrease in the IGL (i.e., there is about 50% of M_4_ MR). Like SCN, other brain areas implicated in biorhythm regulation (SPVZ, PHA) did not reveal an M_4_ MR decrease. These data give evidence of the main role of M_4_ MR in MOCx, SSCx, TH, and IGL in biorhythm regulation rather than in SCN, SPVZ, and PHA. In an important way, IGL can provide feedback regulation (or fine tuning) of locomotor activity influencing SCN (Hughes & Piggins, 2012), and, thus, it should be stressed that its role in M_4_ MR affected locomotor regulation.

Please note that although the density in TH is comparable to the density in SCN, we were able to detect about a one‐half decrease in this area, indicating that we can detect a decrease even if the density in the area is quite low.

As muscarinic receptor subtype expression in the SCN is still a matter of debate, we clearly show here that the number of M_4_ MR in the SCN is inappreciable. The initial paper that tried to detect MR in SCN used also autoradiography (Bina, Rusak, & Wilkinson, 1998). These authors revealed that the muscarinic receptor density in the SCN is very low, mainly when compared to the striatum. We confirm this finding and are adding new knowledge about no M_4_ MR presence in SCN. Another report indicated the presence of MR (generally) using immunohistochemistry (Hut & Van der Zee, 2011). It is not surprising that the PCR technique identified all five muscarinic receptor subtypes in the rat SCN (Yang, Wang, Cheng, Kuo, & Huang, 2010). The number of studies trying to identify the functional role of muscarinic receptor subtype in the SCN is limited. Carbachol, a muscarinic agonist, has been shown to induce phase advance in the circadian rhythm of spontaneous neuronal activity (Gillette et al., 2001), thus indicating the role of MR in the SCN. Taken together with our data, it should be another MR subtype that could be responsible for phase shift in the SCN, which also confirms with conclusion of Gillette et al. (2001), which suggested that this effect is M_1_ MR implicated.

Given the current understanding of M_4_ modulation of dopamine signaling and evidence from M_4_ KO mice, the impaired cholinergic control of dopamine signaling either directly in the striatum (Cachope & Cheer, 2014; Shin, Adrover, Wess, & Alvarez, 2015), or, in a more complex manner, involving polysynaptic circuits (Tzavara et al., 2004), can be suggested as the underlying mechanism affecting the motor activity and biorhythm in M_4_ KO mice. M_4_ MR are particularly abundant in striatum, where they modulate dopamine transmission. The striatum is the main input structure of the basal ganglia circuitry network, processing inputs from several other brain areas including the whole cortical matter (Groenewegen, 2003). Numerous studies have demonstrated acetylcholine–dopamine interactions within the striatum (Cachope & Cheer, 2014). In the recent past, the role of MR in local control of dopamine release in the nucleus accumbens, the ventral striatum, has been clarified. M_2/4_ MR have been shown to decrease the dopamine release triggered by stimulation of nicotinic receptors located at dopaminergic terminals via autoinhibition of ACh release (Shin et al., 2015). In the striatum, M_4_ serve as the main autoinhibitory receptors (Zhang et al., 2002).

We have hypothesized gender differences in motor coordination according to previous data (Kuljis et al., 2013). We have found only marginal changes in males, but clearly pronounced activity changes in females. There are also some other data showing gender differences in the running wheel, light‐dark transition test, elevated plus maze, and open field (Blizard et al., 1975; Morgan & Pfaff, 2001; Ogawa et al., 2003). The effect on locomotor activity is mediated via the estrogen receptor α (Ogawa et al., 2003). Moreover, morphological sex differences have been shown in the volume of the SCN (Gorski, Gordon, Shryne, & Southam, 1978). In an important way, gender differences in the ^3^H‐AFDX‐384 binding sites have been found using autoradiography in striatum, nucleus accumbens, and olfactory tubercle (Fragkouli, Stamatakis, Zographos, Pachnis, & Stylianopoulou, 2006). More specific, although these authors (Fragkouli et al., 2006) described ^3^H‐AFDX‐384‐binding sites as M_2_, they are, in fact, represented by mixed M_4_/M_2_ population as we have described recently (Valuskova et al., 2018). Moreover, in caudate putamen, nucleus accumbens, and olfactory tubercle, 77.7, 74.2, and 74.6% of ^3^H‐AFDX‐384‐binding sites, respectively, are represented by M_4_ MR and M_2_ MR constitute only a minor portion, with majority of binding to M_4_ (Valuskova et al., 2018). Thus, we can consider the autoradiography binding in females as representative to brain areas responsible for locomotor regulation.

In an important way, the sex hormones have been shown to affect M_4_ MR (El‐Bakri et al., 2002). Ovariectomy upregulated M_4_ MR in the hippocampal (dentate gyrus, CA1, CA3), hypothalamic structures, and in the frontal cortex. Estrogen substitution led to restoration of M_4_ MR initial levels. In addition, ovariectomy decreased the exploratory (i.e., locomotor) activity of the rats that were restored by estrogen treatment. This can be hypothetically the reason for biorhythm changes in females: If ovariectomy upregulates M_4_ MR and decreased activity, then M_4_ MR knockout would have contrary effects. Progesterone treatment had no effect on the ovariectomy‐induced upregulation of M_4_ receptors.

Furthermore, some studies proved that circadian rhythmicity can be affected by sex hormones (Bailey & Silver, 2014), which are, per se, also the subject of rhythmicity. In the 80s, Wollnik (1985) observed obvious sex differences in the daily pattern of locomotion in laboratory rats. Hormonal and genetic differences between males and females also influence development of locomotor activity circadian rhythm (Diez‐Noguera & Cambras, 1990). In the same way, estradiol has been shown to influence the level and distribution of daily locomotor activity, the response to light pulses behavior, and the time span of the free‐running period (Blattner & Mahoney, 2014). The nature of sex differences is not clear to date but hypothetically can also arise from higher androgen receptor (AR) expression in the SCN in males (Bailey & Silver, 2014).

Taking these data together with our results, we can conclude that non‐SCN M_4_ MR play a role in motor activity biorhythm regulation and that the IGL, together with the striatum and MOCx, is suspicious areas involved in this regulation.

## CONFLICT OF INTEREST

The authors declare that the research was conducted in the absence of any commercial or financial relationships that could be construed as a potential conflict of interest.
