# Supplementary material for: The deletion of M4 muscarinic receptors increases motor activity in females in the dark phase
Source: Brain Behav. 2018 Jul 6;8(8):e01057. doi: 10.1002/brb3.1057 (PMC6085911; doi:10.1002/brb3.1057)
Supplement: Supplementary file 3 [file BRB3-8-e01057-s003.docx]

**Supplementary table 1:** Differences in activity biorhythm parameters between WT and M_4_KO males. The data were analyzed using one-way ANOVA with post-hoc Sidak's corrections or using Student t-test in parameters that do not reveal correlations (WT vs. KO animals). *p<0.05, **p<0.01, ***p<0.001. For explanation of biorhythm parameters see Table 2.

| Parameter | T Highest | D Mean | D Highest | N Highest | P lengths | T AUC | Max Slope | Min Slope |
| --- | --- | --- | --- | --- | --- | --- | --- | --- |
| significance | ** | * | * | * | * | *** | *** | *** |
| **% KO *vs* WT** | **111.22** | **118.98** | **114.14** | **109.52** | **74.19** | **110,30** | **1.53** | **1.53** |

| **Supplementary table 2:** Differences in temperature biorhythm parameters between WT and M_4_KO males. The data were analyzed using one-way ANOVA with post-hoc Sidak's corrections or using Student t-test in parameters that do not reveal correlations (WT vs. KO animals). *p<0.05, **p<0.01, ***p<0.001. For explanation of biorhythm parameters see Table 2. *p<0.05, ***p<0.001.   \| Parameter \| T Lowest \| D Lowest \| N Lowest \| Through \| Amp 12 \| \| --- \| --- \| --- \| --- \| --- \| --- \| \| significance \| * \| * \| * \| * \| *** \| \| **% KO *vs* WT** \| **99.37** \| **99.40** \| **99.08** \| **99.51** \| **242.79** \| |  |
| --- | --- | --- | --- | --- | --- | --- | --- | --- | --- | --- | --- | --- | --- | --- | --- | --- | --- | --- | --- |

**Supplementary Table 3:** Differences in temperature biorhythm parameters between WT and M_4_KO females. The data were analyzed using one-way ANOVA with post-hoc Sidak's corrections or using Student t-test in parameters that do not reveal correlations (WT vs. KO animals). For explanation of biorhythm parameters see Table 2. *p<0.05, **p<0.01.

| Parameter | D Highest | N AUC | Max Slope | Amp 12 |
| --- | --- | --- | --- | --- |
| significance | * | * | ** | * |
| **% KO *vs* WT** | **100.49** | **99.34** | **340.05** | **153.76** |

**Supplementary table 4:** Differences in temperature biorhythm parameters between WT males (M) and females (F). The data were analyzed using one-way ANOVA with post-hoc Sidak's corrections or using Student t-test in parameters that do not reveal correlations (WT vs. KO animals). For explanation of biorhythm parameters see Table 2. *p<0.05, **p<0.01, ***p<0.001.

| Parameter | T AUC | T Highest | T Lowest | D Mean | D Highest | N Highest | Trough | Peak | Acro 24 |
| --- | --- | --- | --- | --- | --- | --- | --- | --- | --- |
| significance | ** | ** | *** | *** | * | ** | *** | *** | * |
| **% F *vs* M** | **114.57** | **101.01** | **102.14** | **101.60** | **100.82** | **101.05** | **101.75** | **102.01** | **102.41** |

**Supplementary table 5:** Differences in temperature biorhythm parameters between KO males (M) and females (F). The data were analyzed using one-way ANOVA with post-hoc Sidak's corrections or using Student t-test in parameters that do not reveal correlations (WT vs. KO animals). For explanation of biorhythm parameters see Table 2. *p<0.05, **p<0.01, ***p<0.001.

| Parameter | T AUC | T Highest | T Lowest | D Mean | D Highest | N Highest | Trough | Peak | Amp 24 |
| --- | --- | --- | --- | --- | --- | --- | --- | --- | --- |
| significance | ** | *** | *** | *** | * | *** | *** | *** | * |
| **% F *vs* M** | **87.40** | **98.71** | **98.27** | **98.73** | **99.43** | **98.71** | **98.63** | **97.41** | **76.76** |
| Parameter | Amp 12 |  |  |  |  |  |  |  |  |
| significance | * |  |  |  |  |  |  |  |  |
| **% F *vs* M** | **66.83** |  |  |  |  |  |  |  |  |

**Supplementary figure 1:** The periodograms in females, showing no differences in WT and KO animals. The spectrum was calculated by Chronos-Fit using Lomb-Scargle algorithm.

**Supplementary figure 2:** Nissl staining (above) and autoradiogram (bottom) showing the localization of suprachiasmatic nucleus (SCN).
